# Tolerance and reward equity predict cooperation in ravens (*Corvus corax*)

**DOI:** 10.1038/srep15021

**Published:** 2015-10-07

**Authors:** Jorg J. M. Massen, Caroline Ritter, Thomas Bugnyar

**Affiliations:** 1 University of Vienna, Department of Cognitive Biology, Vienna, Austria; 2 Haidlhof Research Station, University of Vienna and University of Veterinary Medicine, Bad Vöslau, Austria

## Abstract

Cooperative decision rules have so far been shown experimentally mainly in mammal species that have variable and complex social networks. However, these traits should not necessarily be restricted to mammals. Therefore, we tested cooperative problem solving in ravens. We showed that, without training, nine ravens spontaneously cooperated in a loose-string task. Corroborating findings in several species, ravens’ cooperative success increased with increasing inter-individual tolerance levels. Importantly, we found this in both a forced dyadic setting, and in a group setting where individuals had an open choice to cooperate with whomever. The ravens, moreover, also paid attention to the resulting reward distribution and ceased cooperation when being cheated upon. Nevertheless, the ravens did not seem to pay attention to the behavior of their partners while cooperating, and future research should reveal whether this is task specific or a general pattern. Given their natural propensity to cooperate and the results we present here, we consider ravens as an interesting model species to study the evolution of, and the mechanisms underlying cooperation.

Cooperation is an important part of human social life, and consequently, has been the topic of extensive cognitive research^1^,^2^. For example, cooperation enhances the development of cognitive abilities in human children^3^. To study the evolution of cooperation a comparative approach is warranted^4^, and a strong focus should lie on the similarities and differences regarding the cognitive and social mechanisms of human and animal cooperation^5^. Field observations have shown animal cooperation within a variety of different social contexts^6^. For example, cooperative hunting has been described in social carnivores^7^,^8^,^9^,^10^, chimpanzees, *Pan troglodytes*^11^, killer whales, *Orcinus orca*^12^,^13^, Harris’ hawks, *Parbuteo unicinctus*^14^, brown necked ravens, *Corvus rufficollis*^15^, and within- and even between different species of fish^16^,^17^. Additionally, social support within agonistic interventions has been extensively studied in many primate-18 and other social species (e.g., ring-tailed coatis, *Nasua nasua*^19^; bottlenose dolphins, *Tursiops truncatus*^20^, fallow deer, *Dama dama*^21^, spotted hyenas, *Crocuta crocuta*^22^, common ravens^23^). Whereas some sophisticated cognition may be attributed to for example the cooperative hunting of chimpanzees^24^ and fish^25^, controlled experiments are warrEnted to determine the specific proximate mechanisms underlying animal cooperation.

Experimental studies on animal cooperation have focused on questions regarding general- and social cognition; i.e., what do the animals understand of the cooperative problem at hand, and what might be social constraints for cooperation. Regarding the former question, most emphasis has been put on whether animals understand the need and role of the partner in cooperation. This understanding is commonly tested using cooperative pulling tasks comparing solitary and dyadic conditions, or with delay trials, where the focal animal has to wait for its partner to arrive before successful cooperation can be achieved. So far, orangutans, *Pongo pygmeus*^26^, capuchin monkeys, *Cebus apella*^27^, cotton-top tamarins, *Saguines oedipus*^28^, chimpanzees^29^, spotted hyenas^30^, Asian elephants, *Elephas maximus*^31^, domestic dogs, *Canis familiaris*^32^, and coral trout, *Plectropomus leopardus*^33^, have been reported to pass these tests; i.e. they differentiated between a cooperative and solitary setting depending on the availability of a partner, and waited for the partner to come if it was delayed. Moreover, the studies by Melis *et al.*^29^ and Vail *et al.*^33^ showed that chimpanzees and coral trout, respectively, are also able to assess the effectiveness of different partners and choose the best one to collaborate with. In contrast, capuchin monkeys in other studies^34^,^35^, baboons, *Papio papio*^36^, tonkean and rhesus macaques, *Macaca tonkeana & M. mulatta*^37^, rooks, *Corvus frugilegus*^5^, and African grey parrots, *Psittacus erithacus*^38^, did not pass the delay and/or solitary control conditions, suggesting that they do not pay attention to the other’s role or even lack an understanding of the need of a partner. Nevertheless, all these animals did spontaneously and successfully cooperate in the experimental task, suggesting that they can achieve cooperation through acting apart together^39^, most probably motivated by a mutual attraction to the apparatus and the food^37^. Moreover, the distinction between species that do or do not understand the need and role of a partner is not that clear-cut, because: a) results are sometimes inconclusive per species (e.g.^27^ vs.^5^,^34^ vs.^32^,^40^); b) the experimental set-ups as well as the criteria of many of these experiments differ substantially^28^,^39^; and c) in some studies it remains unclear whether the passing of the controls can be ascribed to true understanding or rather to shaping by extensive training^5^,^41^,^42^.

Regarding social constraints, several studies have now shown that inter-individual tolerance is an important factor explaining the likelihood and success of cooperation^5^,^30^,^43^,^44^,^45^,^46^. This works in two ways: one being that when a species is very tolerant, or at least more tolerant than another species, this makes it more likely to cooperate^45^. Secondly, that specific tolerant dyads of a normally relatively intolerant species cooperate more and are more successful while cooperating^5^,^30^,^43^,^44^,^46^. Whereas the former is a more general species-specific pattern, the latter is flexible; i.e., an individual can tolerate another or not on the basis of its experience in preceding interactions^39^.

Historically, most of the experimental work on cooperation has focussed on socially complex mammal species and so far only two bird species, rooks and African grey parrots, were tested. Interestingly though, both species did show partner choice based on inter-individual tolerance, yet failed the control tests that specifically tested for the understanding of the role of that chosen partner in the cooperative act^5^,^38^. Seed and colleagues^5^ argue that evolutionary differences in understanding between rooks and, for example, chimpanzees might be due to their socio-ecology; i.e. whereas both species do form alliances, chimpanzees live in a much more complex social environment than rooks. Therefore, it would be interesting to study the cooperative solving abilities of a bird species that lives in a similarly complex social environment as chimpanzees do; i.e., high degree of fission-fusion dynamics and multiple differentiated social relationships to various group members.

Like in rooks, the main unit of raven sociality is the long-term pair bond between the breeding male and female, which also functions as an alliance in conflicts with conspecifics^47^. Unlike rooks, breeding is restricted to those pairs who succeed in defending a territory; depending on the availability of suitable territories, ravens thus experience a prolonged period as non-breeders (which may extend up to 1/3 of their expected life span)^48^. Non-breeding ravens form groups during foraging, roosting and socializing, with group size ranging from a few to several hundreds or even thousands of birds^49^, and with the composition of group members changing during the day and across contexts^49^,^50^. Despite these high degrees of fission-fusion dynamics, temporarily stable sub-groups can be observed, i.e. certain birds use the same food source/roost repeatedly, over consecutive weeks or years^50^. Moreover, individuals form multiple highly differentiated affiliative and agonistic relationships with others that comprise qualities^51^ comparable to those of primates^52^. Hence, raven non-breeder groups show the above-mentioned characteristics of high social complexity.

Recent studies have revealed that ravens, similar to for example chimpanzees, also exhibit the socio-cognitive traits necessary to maneuver successfully through such a complex social world^53^: i.e., ravens have been shown to deceit others while caching food^54^, reconcile with- and console valuable partners^55^,^56^, remember former group mates and their relationship with them over years^57^, understand third-party relations^58^, and use such understanding to prevent others from forming too strong alliances^59^. Moreover, ravens also show cooperation in the wild; e.g. they support each other in conflicts^23^, hunt cooperatively^60^, and cooperatively chase away larger predators or dominant pairs from prey items^61^,^62^.

We investigated the cooperative problem solving abilities of ravens by using a set-up comparable to most other studies based on the loose string paradigm^63^. We investigated these abilities in a group of captive non-breeding ravens in two different social contexts: In experiment 1, seven ravens were confronted with the string pulling set-up in a group setting, which has been considered as a more flexible^64^ and ecologically more relevant^65^ situation than a setting with pre-arranged dyads because individuals can choose when to cooperate and with whom^45^,^65^,^66^. In experiment 2, we tested the same seven, plus two additional ravens in the more controlled dyadic set-up, and tested all possible (=36) dyads. This dyadic set-up allowed us to test in more detail the effect of specific inter-individual characteristics on cooperation like social relationship quality and reward distribution. Moreover, in the dyadic set-up, we also implemented two control conditions i.e., solitary- and delay trials, to test the ravens’ understanding of the need for a partner. Finally, we ran co-feeding experiments before, in between, and after the two cooperation experiments, in which we tested which individuals feed next to each other at the apparatus (without the necessity to cooperatively pull one string). Subsequently, we used these data as a proxy for tolerance in the analyses of our cooperation experiments (see Additional Methods for a more detailed description of these experiments).

## Study 1

### a. Methods

**Figure 1:**
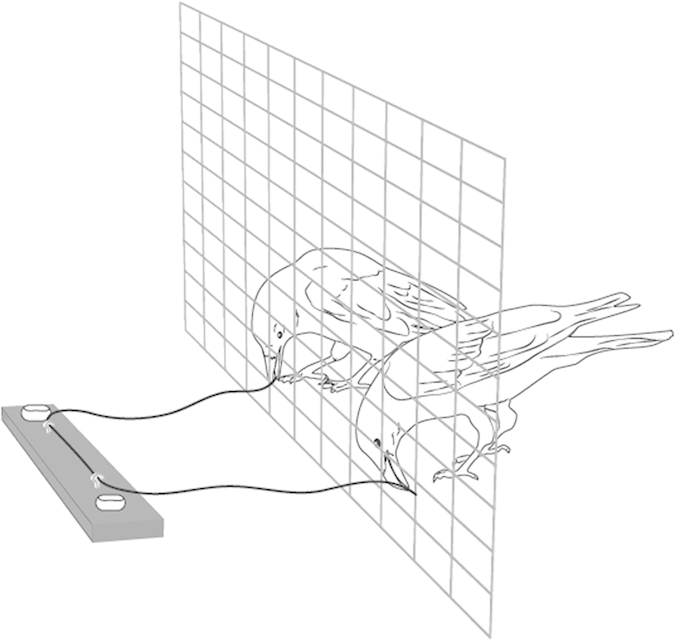
Experimental set-up. Two birds have to pull the two ends of the string simultaneously to move the feeding platform in reach. If only one bird pulls, the string will just go through the two metal loops anchored to the feeding platform and become unthreaded, while the platform remains stationary. Picture drawn by Nadja Kavcik-Graumann.

7 subadult ravens (*Corvus corax*) living at the Haidlhof research station in Bad Vöslau, Austria, participated in this study (see electronic supplementary materials). We used a loose-string set-up (cf.^63^); i.e., a feeding platform (78 cm × 10 cm × 1,0 cm) was placed on top of a larger wooden platform (200 cm × 60 cm × 1,5 cm) outside the aviary. The larger platform was at the same level as the inner ground floor of the aviary. The feeding platform was always baited with two pieces of cheese (±5 mm^3^) 70 cm apart from each other at the front of the platform. Two metal loops were anchored to the feeding platform, and a string was threaded through the metal loops. Both ends of this string then were placed in the aviary, and only if both ends were simultaneously pulled, the feeding platform would move towards the aviary. If only one end of the string would be pulled, the string would become unthreaded and the platform would stay stationary (Fig. 1). The experimenter would then quickly secure and retrieve the string, and end the trial. We choose to secure and retrieve the string because from previous experiences we know that these ravens like to play with strings, and we wanted to avoid that unsuccessful trials were somehow rewarded.

All individuals (*n* = 7) had simultaneous access to the apparatus. The group received a total of 600 trials divided over 30 sessions (20 trials each); the 30 sessions were divided over 10 different days (ranging between 2 to 9 session/day), with at least 5 minutes between each session. At the start of each trial, the experimenter called for the attention of the ravens, baited the feeding platform and then placed the two ends of the string into the aviary. A trial would end when either two birds successfully cooperated, one bird pulled alone and unthreaded the string, or when two minutes had passed. Inter-trial interval was 20 s.

Per trial we coded which individual pulled on the end of a string, be it solitary or while someone else was also pulling on the other side, and thus also whether they cooperated successfully or not. In addition, data regarding the dominance hierarchy within the group and inter-individual tolerance were obtained throughout the testing period. (For more detailed procedures see Additional Methods below)

### b. Results

**Figure 2:**
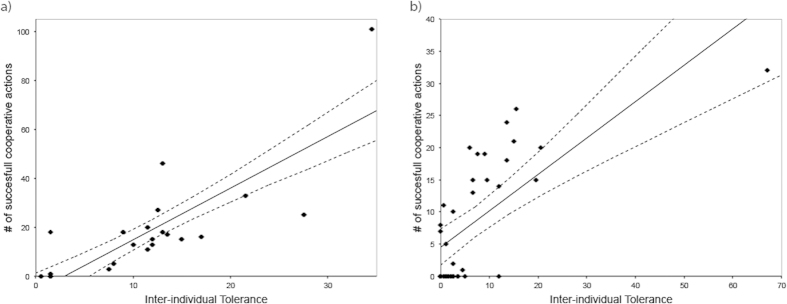
(**a**) Mean inter-individual tolerance score per dyad (see ESM) and its relation to cooperative success in the group setting (study 1), and (**b**) mean inter-individual tolerance score per dyad (see ESM) and its relation to cooperative success in the dyadic setting (study 2). Solid lines reflect trend-lines and dashed lines indicate the 95% confidence intervals.

The ravens cooperated successfully in 397 out of 600 trials (66.17%). Every individual was successful in at least 32 trials, yet there was quite some variation in how successful they were with all possible partners, and 3 out of 21 possible dyads never cooperated successfully with each other. To test what possible factors might explain the difference in success between the different dyads, we ran a generalized linear mixed model (GLMM) on the number of successful cooperation trials, in which we added inter-individual tolerance levels, dominance rank, rank distance, sex, sex-combination and kinship (yes/no) as fixed effects, and the identity of the dyad and the subject of analyses as random effects to control for repeated measurements. We found that inter-individual tolerance was the only significant predictor of successful cooperation (*β* = 1.810, *F* = 13.2, d.f.n. = 1, d.f.d. = 34, *P* = 0.001), suggesting that cooperative success increases with increasing inter-individual tolerance (Fig. 2a) (see electronic supplementary materials for best fitting model).

## Study 2

In study 1, the highest-ranking individuals could and partly did monopolize the apparatus until they were satiated. Consequently, not all dyads had the full opportunity to cooperate with each other equally often. Therefore, in study 2 the subjects were tested in a dyadic set-up, and all possible dyads were tested twice. Furthermore, in this more controlled set-up, we also conducted two controls to test for the ravens’ understanding of the need of a partner in this task.

### a. Methods

The same 7 subjects as in study 1 and two additional subjects participated in study 2 (*n* = 9). In addition, the same apparatus and set-up as in study 1 was used.

All individuals were tested with all other individuals in two sessions; i.e., we tested all 36 dyads in two rounds, and per round the order of dyads was randomized. Per session, each dyad received 16 cooperation trials interspersed with per individual 2 solitary and two waiting trials. Over the 2 rounds of 36 sessions/dyads, in sum each dyad received 32 cooperation trials, and each individual received a total of 32 solitary- and 32 waiting trials.

In the solitary and waiting trials we tested whether the ravens had an understanding of the need for a partner; i.e. whether they could inhibit pulling and wait for a partner to arrive and to cooperate with them. In a solitary trial one individual was presented with both ends of the string, yet without a partner present in the same compartment. In a waiting trial again one individual was solitary, but as soon as the two ends of the string were placed in the aviary, a door (at a distance of 2.5 meter from the apparatus) was opened, allowing also the second individual access to the room with the apparatus. In order to successfully cooperate in such a waiting trial, individual A thus had to wait for individual B to arrive before pulling (cf.^5^). Both solitary and waiting trials were ended after two minutes, when the focal bird pulled the rope by itself or, in the case of the waiting trials when the focal bird waited and then cooperated successfully with its partner.

The order of events/trials in a session was as follows: At the start of the session two birds were separated from the group and were placed in two adjacent compartments, separated from each other by wire-mesh; Individual A received 2 solitary trials; Individual A received 1 waiting trial; The dyad received 4 cooperation trials; Individual B was again separated in the adjacent compartment; Individual A received 1 waiting trial; The dyad received 4 cooperation trials; Individual A was separated in the adjacent compartment; Individual B received 2 solitary trials; Individual B received 1 waiting trial; The dyad received 4 cooperation trials; Individual A was again separated in the adjacent compartment; Individual B received 1 waiting trial; The dyad received 4 cooperation trials.

We coded how often the individuals in a dyad cooperated successfully, i.e. pulled on both ends of the string simultaneously, and if so whether they divided the rewards equally; i.e., did both birds take 1 piece of cheese, or did one bird monopolize the feeding platform after cooperating and take both pieces of cheese. In the solitary trials we coded whether an individual pulled the string or not, and in the waiting trials, we coded whether an individual waited with pulling until its partner had arrived (for full coding and more detailed procedures see Additional Methods below).

### b. Results

The ravens cooperated successfully in 314 out of 1152 trials (27.26%). Every individual was successful at least once, yet there was again quite some variation in how successful they were with all possible partners, and 15 out of 36 possible dyads never cooperated successfully with each other. In general, the birds performed significantly worse in the dyadic tests than in the group test (study 1), as the proportion of successful cooperation trials in the latter was significantly higher than that of the former (*χ*^2^ = 675.8, df = 1, *P* < 0.001). To test what possible factors might explain the difference in success between the different dyads, we ran a GLMM on the number of successful cooperation trials, including the same factors as in study 1. In addition, here we also included session number/individual (ranging between 1–16) to see whether there were any learning effects. We found that session number, sex combination of the dyad, dominance rank distance of the dyad, kinship and inter-individual tolerance, all had a significant effect (*P* ≤ 0.05) on the number of successful cooperation trials (see electronic supplementary materials for best fitting model). The number of successful cooperation trials increased significantly with increasing session number (*β* = 0.176, *F* = 26.25, d.f.n. = 1, d.f.d. = 137, *P* < 0.001), suggesting a learning effect. However, follow up analyses showed that also the total number of pulls (be it cooperative or unsuccessful solitary pulls) significantly increased with increasing session number (*β* = 0.250, *F* = 9.17, d.f.n. = 1, d.f.d. = 142, *P* = 0.003), suggesting that the increase in successful cooperation over time was rather a motivational issue than a learning effect. Regarding the effect of sex-combination, post-hoc analyses revealed that mixed-sex dyads performed significantly better than male-male dyads and a similar trend was found with regard to female-female dyads (mm: μ = 4.73 (out of 32) ± s.e.m. 1.71; mf: μ = 14.90 ± s.e.m. 2.67; ff: μ = 8.0 ± s.e.m. 2.84; Wilcoxon Signed Rank tests: mm/mf: *T*^+^ = 21.0, *n* = 6, *P* = 0.028; mf/ff: *T*^+^ = 6.0, *n* = 3, *P* = 0.109; Mann Whitney U; mm/ff: *U* = 13.0, *n* = 6/3, *P* = 0.381). Regarding the effect of dominance rank distance, the number of successful cooperation trials increased with increasing dominance rank distance (*β* = 0.331, *F* = 5.60, d.f.n. = 1, d.f.d. = 137, *P* = 0.019), suggesting that individuals that are not close to each other in the dominance hierarchy cooperate better than those that are close to each other in the dominance hierarchy. Regarding the effect of kin, post-hoc analyses revealed that kin cooperated significantly more than non-kin (Kin: μ = 18.86 (out of 32) ± s.e.m. 1.31; Non-kin: μ = 7.24 ± s.e.m. 1.29; Wilcoxon Signed Rank tests: *T*^+^ = 28.0, *n* = 7, *P* = 0.018). Finally, inter-individual tolerance had a significant positive effect on the number of successful cooperation trials (*β* = 0.133, *F* = 35.10, d.f.n. = 1, d.f.d. = 136, *P* < 0.001), corroborating our findings from study 1 (Fig. 2b).

**Figure 3:**
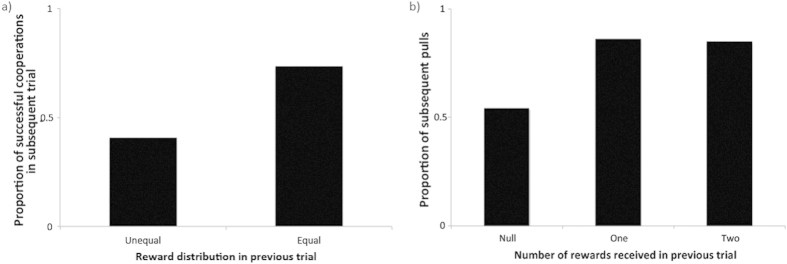
(**a**) Proportion of trials in which two birds cooperated successfully subsequent to an equal or unequal reward devision after the previous successful cooperation trial, and (**b**) proportion of trials in which a bird pulled the string after it had received zero, one or two rewards in the previous successful cooperation trial.

The experimenter always placed 1 piece of cheese on both ends of the feeding platform to allow both collaborators to retrieve one piece after cooperating. However, sometimes one individual would quickly take both rewards after a successful cooperation, leaving its collaborator without reward. To see whether the birds reacted to the reward distribution after a successful cooperation trial, we analyzed for all trials, after the first successful cooperation trial within a session, what would be the probability that both birds would cooperate successfully again. Therefore, we ran a binomial GLMM with a logit link function on whether a dyad would cooperate (yes/no), and entered the same fixed factors as previous analyses, although now we treated session number as a random variable. In addition, we now entered whether the reward distribution in the previous successful trial was equal (yes/no) as a fixed factor. We found that in the best fitting model only inter-individual tolerance still had a positive effect on the probability that two birds would cooperate successfully (*β* = 0.107, *F* = 15.31, d.f.n. = 1, d.f.d. = 889, *P* < 0.001), whereas all previous significant effects were now not significant anymore, and that indeed the reward equity of the previous successful cooperation trial within a session had a significantly positive effect on the probability that two birds would cooperate successfully (*β* = 0.804, *F* = 12.98, d.f.n. = 1, d.f.d. = 889, *P* < 0.001) (for best fitting model see electronic supplementary materials); i.e., if the reward distribution in the previous successful trial was equal (1/1) the probability of two birds successfully cooperating again was significantly higher than when the reward distribution in the previous successful trial was unequal (2/0) (Fig. 3a). This would suggest that birds that did not receive a reward in the previous successful cooperation trial, are less motivated to cooperate again. Indeed the amount of rewards a bird received in the previous successful cooperation trial (0, 1, or 2) significantly influenced the probability that a bird would pull at all in the next trial (be it cooperative or unsuccessful solitary pulls) (*F* = 18.03, d.f.n. = 1, d.f.d. = 889, *P* < 0.001); i.e., birds were significantly less likely to pull when they received no reward in the previous successful cooperation trial then when they had received either 1 or 2 rewards (Fig. 3b). To determine whether the bird that previously got all the food in a trial (i.e. ‘cheated’ in the cooperation) would respond to the declined motivation of its partner, we compared the proportion of ‘cheats’ (i.e., taking both rewards instead of sharing) after a previous cheat with the proportion of cheats in general. We found that after cheating the cheater did not become more fair to get back the motivation of its partner, but instead became even more likely to cheat again (*χ*^2^ = 6.79, d.f. = 1, *P* = 0.009), suggesting that if they had learned anything, then it was how to cheat.

With regard to the control trials, in 243 out of 288 solitary trials (84.38%) individuals pulled the string even though this had no effect. Similarly, only 2 birds waited in 5 (out of 288: 1.73%) waiting trials for their partner to arrive at the apparatus and subsequently cooperated successfully. These data suggest that the ravens did not understand the need for a partner to solve the task in this experimental set-up. Moreover, they also showed no sign of learning to wait as they even increased their pulling rate in the solitary trials from the first round of sessions to the second round (Wilcoxon Signed Ranks test: *T*^+^ = 32, *n* = 9, *P* = 0.04), most probably due their general increase in motivation during the study (see above).

## Discussion

Our results show that ravens can spontaneously solve the loose string paradigm; i.e. without any training. Moreover, ravens do not cooperate indiscriminately with everybody, and in both study 1 and study 2 we could demonstrate that inter-individual tolerance is very important for successful cooperation. Specifically, ravens cooperate more with those individuals with whom they have high inter-individual tolerance than with those whom they do not tolerate/are not tolerated by. Even though they attend to who is sitting next to them, they do not seem to attend to what this individual is doing. In particular, in study 2 we found no proof that the ravens understood that actions from their partner were required for cooperation to be successful. However, the ravens did seem to pay attention to and act upon the outcome of a cooperative interaction, as in study 2 we showed that the reward distribution in a previous interaction influences whether two birds will cooperate again. Particularly, chances for renewed cooperation were higher when the reward division in the previous trial was equal.

Inter-individual tolerance was the best predictor for cooperative success. This finding corroborates results in several primate species^37^,^43^,^44^,^45^,^46^, hyenas^30^ and in other bird species^5^,^38^, suggesting a general pattern among social animals. Our study, however, strongly adds to this argument by showing this effect not only in forced partner combinations, but also in a group setting (see also^46^). Whereas the former may allow better titrations of separate variables, the latter represents a much more ecologically relevant setting in which individuals can choose themselves with whom they want to cooperate. This free choice of preferred cooperation partners with high inter-individual tolerance may then also explain why the birds in general performed better in the group setting (study 1) than in the dyadic setting (study 2).

Like chimpanzees^46^, for the ravens also kinship enhanced cooperative success, most probably due to reduced competition over access to the strings and rewards. We think reduced competition may also explain the positive effect of rank distance and the better performance of mixed-sex dyads. In particular, we think that the larger the difference in dominance rank, the fewer direct competition is at play, which holds for the effect of dominance rank difference, but also for the mixed sex dyads, as in ravens males outrank females and females are thus not competitors of males^50^. This finding, however, contrast with findings on chimpanzees that cooperate better with individuals that are close in rank to themselves^46^, but these authors also suggest that this might be due to reduced competition. This contrast becomes particularly interesting when put in the perspective of human cooperation, where females indeed rather cooperate with other females close in rank, whereas males do not attend to the rank of other males when cooperating^67^. It should be noted, however, that we could not replicate the differences between kin and sex-combinations and the effect of rank distance in the group setting, and thus, that these effects may be less pronounced when individuals are not forced into a dyad.

Although the experimenter in our study placed the two rewards within the dyadic setting with ‘the intention’ of an equal reward division, sometimes one of the two birds got two rewards, whereas the other got none. This reward division can come about through two processes, either one bird displaces its collaborator and grabs both rewards, or one bird yields and allows its collaborator to grab both rewards. The latter was not the case in our study, corroborating recent experimental findings that report a lack of prosociality in ravens^68^,^69^. Unequal reward divisions were always due to displacements, mostly by the highest-ranking individual of the two. This creates an asymmetrical situation with ‘cheaters’ gaining the reward for cooperation, whereas those ‘being cheated on’ are left without a reward. Note that in our iterated set-up, however, the latter may have some form of partner control by defecting in subsequent interactions (cf.^39^,^66^).

Indeed, from the perspective of the ‘being cheated on’ individuals, our study is the first to show that a bird species’ behavior is influenced by the behavior of its partner in trial x-1 in an iterated cooperative problem in a relatively natural setting. In particular, ravens where less likely to cooperate when they had been ‘cheated’ on in the previous successful cooperation trial. In contrast, ‘cheaters’ remained motivated to pull (Fig. 3b). This result suggests that these ravens’ choices to cooperate or to defect follow some sort of tit-for-tat rule, contingent even in the short term^70^. This corroborates findings in either a one-shot natural situation in pied flycatchers, *Ficedula hypoleuca*^71^, or in a rather artificial, yet iterated situation in blue jays, *Cyanocitta cristata*^72^, and also corresponds to recent findings in chimpanzees^70^. From a proximate perspective, this pattern may be achieved through some sort of attitudinal reciprocity in which by cheating a negative attitude towards that individual emerges in the other (cf.^73^, but for an elaborate discussion of proposed proximate mechanisms of reciprocity also see^39^). Alternatively, this pattern may be obtained by associative learning, where a negative experience leads animals not to act anymore. Such an explanation, however, would exclude any partner specificity, whereas our results show that irrespective of the reward equity effect, inter-individual tolerance still plays an important role; i.e. even though in general birds become less likely to cooperate after being cheated on, those that are paired with a friend resume cooperating earlier than those that are paired with a non-friend.

From the perspective of the ‘cheater’, obviously cheating itself is the optimal strategy, as reflected in their continuing motivation to ‘cooperate’ after cheating, unless of course the collaborator who is ‘being cheated on’ subsequently starts defecting. Although those ‘being cheated on’ indeed started to defect, the ‘cheaters’ did not seem to detect these defections, or at least did not change their behavior as a result. Specifically, they were not more likely to divide the rewards equally after their collaborators defected, but rather were more likely to ‘cheat’ again. Again, a learning mechanism may be at play in which the birds learn that ‘cheating’ is beneficial, and since the defection rates were rather variable, they just always try to cheat whenever they get the opportunity. Therefore, more controlled experiments in which defection rates can be manipulated are needed to precisely study the proximate mechanisms at hand in such an asymmetrical two-player game.

Finally, the results of this study suggest that ravens do not understand the need of a partner while cooperating, which would put the ravens in line with rooks^5^ and several monkey species^34^,^37^, yet in contrast to apes^26^,^29^,^45^,^46^, capuchin monkeys^27^, cotton-top tamarins^28^, hyenas^30^, dogs^32^, Asian elephants^31^ and coral trout^33^. This finding is rather surprising, since wild ravens flexibly adjust their behaviour depending on their need of a cooperation partner in competition for food^61^,^62^,^74^. Note, however, that the comparison of these studies is difficult since set-ups differ, and whereas some species were heavily trained to, for example, wait in the paradigm (e.g.^29^), others waited spontaneously (e.g.^31^). It is possible that the apparent lack of understanding in the current study is specific for the paradigm used. It seems that the ravens had problems with inhibiting to pull when the partner was absent, a result also found, albeit to a lesser degree, in chimpanzees^70^ and dogs^32^. Possibly our method of the experimenter securing the string before it could be pulled through both loops by a single subject exaggerated this effect and led the ravens to focus on the action of the experimenter (being quicker in pulling) rather than on its partner (waiting for him to come). Future research should reveal whether ravens in general do not understand the need for a partner while cooperating, or whether it depends on the paradigm used and the experience they have with this paradigm.

## Additional Methods

### Subjects, their housing and the period of data collection

Subjects were 9 sub-adult common ravens (6 males, 3 females) of our captive colony at the Haidlhof research station of the University of Vienna and of the Veterinary University of Vienna, in Bad Vöslau, Austria. At the time of testing, the birds were housed in one social group made up of 10 individuals that were derived from four nests in captivity (Stockholm, Bayrischer Wald, Wels and Haag). All birds arrived at Haidlhof research station in 2012 as nestlings at the age of 3 to 5 weeks, were hand-raised to fledging and kept in this social group ever since. They were housed in a large outdoor aviary complex (15 × 15 × 5 m) composed of several compartments. The compartments could be closed off by sliding doors and used for separating of one or more individuals from the group for testing. All birds were familiarized with the separation procedure shortly after fledging using positive reinforcement. They were never food deprived and fed a diet consisting of meat, milk products, bread, vegetables and fruits twice daily; water was available ad libitum. Participation in experiments was voluntary, with birds coming in after being called by their name and in expectation of small rewards not included in their daily diet. In the current studies, the lowest ranked male did not participate in any of the tests and therefore was excluded from all analyses. Two other subordinate males (Paul, Rufus) participated only in Study 2 (see Supplementary Table 1), since continuous and consistent conflicts between them and other group members led us to temporarily separate them from the rest of the group, together with the other subordinate bird. Consequently, they could not participate in the group experiment. Fortunately, the relationships between these birds improved over the course of this study, which allowed us to include these two birds in study 2. Study 1 was conducted between September and December 2013, and study 2 between January and March 2014.

### Ethical note

Participation of the birds in our experiments was voluntarily. Since all experiments were non-invasive, the study complied with Austrian law. Moreover, the study received oversight from- and was authorized by the ethical board of the behavioural research group at the faculty of Life sciences, University of Vienna (case number: 2015-003). The birds remained in captivity at the Haidlhof Research Station after the study.

### Procedure and design

#### Familiarization

Even though the set-up was positioned outside of the aviary, all individuals were habituated stepwise to the apparatus, taking possible effects of neophobia towards novel objects into account. The ravens were first introduced to the big panel (200 cm × 60 cm × 1,5 cm) and then to the small panel (78 cm × 10 cm × 1,0 cm) 2.5 weeks before the experiment started. During this habituation phase the whole group had permanent access to the set-up and we regularly placed small treats on the set-up that were in reach for the ravens. We only started testing after we had established that all individuals regularly came to the set-up to take some of the treats.

#### Cooperative string-pulling test (group)

The whole group participated in one round of cooperative group tests in order to asses whether the ravens would solve the cooperative problem spontaneously and to subsequently asses which factors influence cooperative success when individuals are able to choose their cooperation partner among all group members. Per day we conducted 3 to 9 sessions with at least a 1 hour break after every third session took place. Each session consisted of 20 trials with a 20 s inter-trial interval and 5 min inter-session interval. In total we ran 30 sessions.

#### Cooperative string-pulling test (dyads)

We ran two rounds of dyadic cooperation tests, which also included control trials. With these tests, we aimed to investigate which factors might influence cooperative success when individuals are forced in certain dyads, and whether the ravens understood the need for a partner within this paradigm. Every individual was tested with every other individual of the group (36 dyads) in one round, and this was then repeated in the second round. The order of dyads was randomized in both rounds. Each session consisted of 16 cooperation trials with a 20 s inter-trial interval, plus, per individual in the dyad, 4 control trials; i.e., 2 solitary trials and 2 delay trials. In the former, the focal bird was solitary, and we were interested to see whether it would not pull the string, since successful cooperation was impossible. In the latter, the other bird in the dyad was granted access to the testing compartment as soon as both ends of the string were placed in the aviary, and we were interested to see whether the focal bird would wait for its partner to arrive at the apparatus before commencing to pull.

### Coding

All trials were videotaped with 2 cameras. CR coded all trials live and afterwards from the videotapes. In study 1 she coded who pulled on which side of the string and whether it eventually led to successful cooperation; i.e., two individuals had to pull each on one end of the string simultaneously in order to move the panel as far to the fence that the food was in reach. A trial was considered unsuccessful when the string got loose, which means that the other end of the string was pulled back out of the aviary and therefore out of reach for the partner. In study 2 she additionally coded whether individuals pulled or not in the solitary trial, and whether they waited in the delay trials. Moreover, in study 2 she coded whether after successful cooperation the two birds would divide the rewards equally or not; i.e., would they both take the one piece of cheese on their side of the feeding platform, or would one of the birds chase away the other and take both rewards. A research assistant independently recoded 10% of the videos of study 1 and 11.11% of the videos of study 2, and inter-rater agreement was good (Cohen’s kappa = 0.70) and excellent (Cohen’s kappa = 0.93) for both studies respectively.

### Additional data

#### Tolerance data

We tested all individuals’ ability to tolerate other individuals in front of the apparatus at the same time. For this, pieces of Frolic^®^ dog food were attached to two separate strings. Birds thus had to pull on one of the strings to reach the reward, without the need of a cooperation partner. Both ends were simultaneously moved into the aviary and were placed in a way that the ends were either 30 cm, 60 cm or 120 cm apart from each other. Providing these different distances could potentially reveal specific distances in which specific birds tolerate each other’s presence in a food-context. Per day, we ran 3 sessions, each with a different distance. One session consisted of 20 trials with a 20 s inter-trial interval and 5 min inter-session interval. Order of distances was counterbalanced over a total of 6 days. Consequently, we conducted 18 sessions, 6 for each distance, per round. We ran 3 of these rounds: one before study 1, one in between study 1 and 2, and one after study 2. Tolerance data were very consistent over the different distances (ICC: 0.855, *p* < 0.001) and over the different testing rounds (ICC: 0.574, *p* < 0.001), and were therefore lumped. However, since there were additional animals involved in study 2 vs. study 1, for the analyses of study 1, we used combined tolerance data based on the tolerance tests before and after study 1, whereas for the analyses of study 2, we used combined tolerance data based on the tolerance tests before and after study 2.

#### Dominance hierarchy data

To determine the dominance hierarchy in the group, during the study period, we conducted 3 different monopolization experiments in which we placed two highly preferred, but monopolizable food-items in the group for 30 minutes and videotaped the whole session. Afterwards, all unidirectional displacements^75^ were scored, and data were arranged in a matrix with actors in rows and recipients in columns. A dominance order most consistent with a linear hierarchy was determined, calculating Landau’s linearity indices (h’) using MatMan 1.1^76^ and the matrix was reordered to best fit a linear hierarchy^77^,^78^. We found a significantly linear hierarchy in the group (*h*’ = 0.858, *n* = 9, p < 0.001, based on 524 interactions and 5.56% unknown relationships).

### Statistical analysis

#### Study 1

To test what possible factors might explain the difference in success between the different dyads, we ran a generalized linear mixed model (GLMM) on the number of successful cooperation trials, in which we added inter-individual tolerance levels, dominance rank, rank distance, sex, sex-combination and kinship (yes/no) as fixed effects, and the identity of the dyad and the subject of analyses as random effects to control for repeated measurements. We ran a full model and several reduced models using a backward step-wise approach. The best fitting model was chosen based on comparisons of corrected Akaike Information Criteria (cAIC).

#### Study 2

To test what possible factors might explain the difference in success between the different dyads, we ran a GLMM on the number of successful cooperation trials, including the same factors as in study 1. In addition, here we also included session number/individual (ranging between 1–16) to see whether there were any learning effects. In addition, to test whether birds reacted to the reward distribution after a successful cooperation trial, we analyzed for all trials after the first successful cooperation trial within a session, what would be the probability that both birds would cooperate successfully again. Therefore, we ran a binomial GLMM with a logit link function on whether a dyad would cooperate (Y/N), and entered the same fixed factors as previous analyses, although now we treated session number as a random variable. In addition, we now entered whether the reward distribution in the previous successful trial was equal (Y/N) as a fixed factor. And finally, we tested whether the amount of rewards an individual received in the previous successful trial would influence whether it would pull at all (be it cooperative or unsuccessful solitary pulls) in the next trial or not. Therefore, we ran a binomial GLMM with a logit link function on whether a bird would pull or not and only entered the amount of rewards that birds had received in the previous trial as a fixed variable, whereas we included the identity of the dyad and the subject of analyses as random effects to control for repeated measurements, and session number as a random effect to control for learning/motivation effects. We ran full models and several reduced models using a backward step-wise approach. Best fitting models were chosen based on comparisons of corrected Akaike Information Criteria (cAIC).

#### Overall

We used Wilcoxon signed ranks tests and Mann-Whitney U tests for post-hoc comparisons and used a Holm Bonferroni^79^ correction for calculating corresponding p-values when multiple comparisons were made on one data-set. All tests were two-tailed and we set alpha to 0.05.

## Additional Information

**How to cite this article**: Massen, J. J. M. *et al.* Tolerance and reward equity predict cooperation in ravens (*Corvus corax*). *Sci. Rep.* **5**, 15021; doi: 10.1038/srep15021 (2015).

## Supplementary Material

Supplementary Information
