# Supplementary material for: Tolerance and reward equity predict cooperation in ravens (Corvus corax)
Source: Sci Rep. 2015 Oct 7;5:15021. doi: 10.1038/srep15021 (PMC4595729; doi:10.1038/srep15021)
Supplement: Supplementary Information [file srep15021-s1.doc]

Supplementary Material for

**“Tolerance and reward equity predict cooperation in ravens (*Corvus corax*)”**

Jorg J.M. Massen^1^, Caroline Ritter ^1^ & Thomas Bugnyar ^1, 2^

^1^ University of Vienna, Department of Cognitive Biology, Vienna, Austria

^2^ Haidlhof Research Station, University of Vienna and University of Veterinary Medicine, Bad Vöslau, Austria

**Supplementary tables**

**Supplementary Table 1.** Names, sex, dominance rank and kinship (nest 1-4) of the animals involved in study 1 and in study 2.

| **Name** | **Sex** | **Rank** | **Origin** | **Study 1** | **Study 2** |
| --- | --- | --- | --- | --- | --- |
| Laggie | M | 1 | Nest 1 | Y | Y |
| Tom | M | 2 | Nest 1 | Y | Y |
| Paul | M | 3 | Nest 3 | N | Y |
| George | M | 4 | Nest 2 | Y | Y |
| Horst | M | 5 | Nest 2 | Y | Y |
| Rufus | M | 6 | Nest 4 | N | Y |
| Louise | F | 7 | Nest 2 | Y | Y |
| Nobel | F | 8 | Nest 2 | Y | Y |
| Adele | F | 9 | Nest 1 | Y | Y |

**Supplementary Table 2** Best fitting model on cooperative success in a

group setting (Study 1).

|  | ***β*** | **± S.E.** | ***F*** | **df_numerator_** | **df_denominator_** | ***p*** |
| --- | --- | --- | --- | --- | --- | --- |
| **Sex** | -2.27 | 2.50 | 0.82 | 1 | 34 | 0.371 |
| **Sex-Combination**^*^  *male-male*  *male-female* | 5.40  -4.92 | 12.47  13.20 | 0.54 | 2 | 34 | 0.586 |
| **Rank** | -0.45 | 0.44 | 1.06 | 1 | 34 | 0.312 |
| **Rank distance** | -0.20 | 2.24 | 0.01 | 1 | 34 | 0.930 |
| **Kinship** | 5.71 | 7.32 | 0.61 | 1 | 34 | 0.441 |
| **Tolerance** | 1.81 | 0.50 | 13.20 | 1 | 34 | **0.001** |

^*^female-female is set as reference

**Supplementary Table 3** Best fitting model on cooperative success in a

dyadic setting (Study 2).

|  | ***β*** | **± S.E.** | ***F*** | **df_numerator_** | **df_denominator_** | ***p*** |
| --- | --- | --- | --- | --- | --- | --- |
| **Session nr.** | 0.18 | 0.03 | 26.25 | 1 | 137 | **<0.001** |
| **Sex-Combination**^*^  *male-male*  *male-female* | -1.97  -1.00 | 0.87  0.94 | 3.39 | 2 | 137 | **0.037** |
| **Rank distance** | 0.33 | 0.14 | 5.60 | 1 | 137 | **0.019** |
| **Kinship** | 1.21 | 0.61 | 3.91 | 1 | 137 | **0.050** |
| **Tolerance** | 0.13 | 0.02 | 35.10 | 1 | 137 | **<0.001** |

^*^female-female is set as reference

**Supplementary Table 4** Best fitting model on probability of cooperative success subsequent trial in the dyadic setting (Study 2).

|  | ***β*** | **± S.E.** | ***F*** | **df_numerator_** | **df_denominator_** | ***p*** |
| --- | --- | --- | --- | --- | --- | --- |
| **Equal distribution of rewards in previous trial (y/n)** | 0.80 | 0.22 | 13.00 | 1 | 889 | **<0.001** |
| **Tolerance** | 0.11 | 0.03 | 15.31 | 1 | 889 | **<0.001** |
